# Team Networking in Palliative Care

**DOI:** 10.4103/0973-1075.76234

**Published:** 2011-01

**Authors:** Odette Spruyt

**Affiliations:** Peter MacCallum Cancer Centre, East Melbourne, Victoria, Australia

**Keywords:** Palliative care, Team, Networking, Interdisciplinary care, Change management

## Abstract

“If you want to travel quickly, go alone. But if you want to travel far, you must go together”. African proverb. The delivery of palliative care is often complex and always involves a group of people, the team, gathered around the patient and those who are close to them. Effective communication and functional responsive systems of care are essential if palliative care is to be delivered in a timely and competent way. Creating and fostering an effective team is one of the greatest challenges for providers of palliative care. Teams are organic and can be life giving or life sapping for their members.

“If you want to travel quickly, go alone. But if you want to travel far, you must go together”

African proverb

## NEED FOR A TEAM

The delivery of palliative care involves many dimensions of care. Physical symptoms need to be addressed through expert assessment, diagnosis and pharmacological and non-pharmacological strategies. Likewise, psychosocial distress is relieved by similar careful assessment and delivery of care to the patient and their carers, care which may include financial assistance, practical aids, counseling, targeted care of children and ongoing emotional support. Such wide-ranging care is ideally delivered by a multidisciplinary team.

## CREATING A TEAM

The multidisciplinary team may be a dedicated palliative care team consisting of specialist palliative care medical and nursing staff, social worker, physiotherapist, pastoral carer, volunteers, and others. It may also be a “virtual team” which forms around the patient and carer with palliative care needs. A virtual team may consist of the general practitioner, primary specialist, palliative care specialist nurse or doctor, community nurse, local pharmacist, medical practice social worker, school support staff, and perhaps a spiritual carer with whom the patient has a long-standing relationship. Communication is the key to ensuring that such a virtual team is able to function and support the patient and each of the team members, who have been brought together because of the shared responsibility for caring for that patient.[1] Such virtual teams are also often created as an extension of core palliative care teams, as dying, like living, is a community event which touches many lives.

Coordination of such teamwork is critical. Strategies to assist professionals share information and better coordinate care include the teleconference and multidisciplinary team meeting. At such meetings, pertinent patient details are shared and goals of care are refined and documented. Ongoing review and sharing of information between the many partners in care may occur through faxes, emails, phone contacts, shared clinics, and family meetings.

## STRENGTHENING THE TEAM

To be a strong team, palliative care team members need to have a common ideal and understanding of the team role and the contribution each team member makes to achieve successful team outcomes. Time spent exploring the values underpinning both the individual motivations and the collective identity of the team is important. Each team member contributes specific skills, experiences, attitudes, and values to the whole. The team is seen to represent and provide a unified model of care. Team activities such as debriefing, service planning, academic development and socializing together assist the development of a strong team, which is able to sustain and support its members.[4]

## TEAM AS “CHANGE AGENTS”

It is neither possible nor advisable for palliative care teams to provide direct care for all patients with palliative care needs. Therefore, models exist to assist teams to direct their efforts to those patients most in need of specialist care and to provide leadership and education to the individuals and teams with which they come in contact. Within every such clinical encounter there exists an opportunity to model the palliative care approach and act as “change agents.” This term is used in implementation science and quality improvement and refers to clinicians acting as catalysts for change through modeling of the new way of doing things and providing leadership to others. In this way, other clinicians observe and incorporate that practice into their care. For consultative palliative care teams, working alongside other clinicians, there is an educational opportunity in every consultation. Referring teams increase their knowledge and expertise in providing palliation by the case-by-case clinical knowledge sharing and observing the impact of palliative interventions, be they pharmacological or effective communication or the power of teamwork.[1 2]

In addition to the clinical care provided, each team member participates in quality improvement and educational activities. These may include the implementation of end-of-life care pathways to improve care of the dying, introducing pain as the fifth vital sign to improve pain assessment and documentation, development of therapeutic protocols and practice guidelines, clinical updates for ward staff, education of undergraduate and postgraduate students, and awareness raising activities for staff, patients and carers such as World Hospice and Palliative Care day promotional activities.

## NETWORKING WITH OTHER TEAMS TO DELIVER INTEGRATED CARE

Multidisciplinary care involves many teams, with different skills. In our cancer centre, patient care is organized around a tumor stream model, in which medical, surgical, radiation oncology, and palliative care specialists interact to provide coordinated care for the patient and family. Outpatient clinics are conducted with all specialists of the tumor stream on hand to review and determine the best management plan for the patient. Such care is complex to deliver. Integrating palliative care into this model of 11 tumor streams has challenged our small team of 2 fulltime consultants, 2 fulltime nurses, and 3 registrars as it has challenged other small allied health departments in our hospital. One creative response of our team has been to develop an ambulatory care team, known as the Rapid Response Team (RRT), able to review patients in the OPD on the day of attendance at their main oncology clinic appointment. This RRT of nurse practitioner candidate and registrar with consultant backup provides pain and symptom advice, liaison with and linkage to community palliative care services, interim phone advice and support to patients and carers, coordination of palliative aspects of care and thus minimizes the need for additional appointments. This team has been welcomed by other specialists and has led to an invitation to increase our involvement in the sarcoma unit outpatient clinic at which a large number of adolescent and young adult patients are seen. In addition, such availability has helped to overcome the barrier to palliative care referrals, which frequently leads to delayed referral and unnecessarily prolonged poor symptom control. Another way to increase the networking between teams has been to attend the weekly tumor stream multidisciplinary team meetings. Opportunities to collaborate in research and in quality improvement activities are also important. For example, at PeterMacCallum, there is a team established to provide psychosocial care for adolescents and young adults called onTrac@petermac. This team identified a strong referral barrier to palliative care for this age group, both within the clinical teams and the patients/carers themselves. By integrating a palliative care nurse into the onTrac team, this referral barrier was removed, and there resulted a threefold increase in the patients receiving palliative care. This has also increased the expertise of the palliative care team in caring for adolescents and young adults. Such collaborations are of great importance and build on the clinical and professional relationships already in place.

In Australia, there is an increasing importance placed on providing palliative care to patients with diseases other than cancer. Services are working to develop relationships with other specialist areas in particular renal and neurology in some centers in order to facilitate patient referral and appropriate care. Learning from specialist colleagues is important to ensure that palliative care decision making is up to date with current expertise in the management of these illnesses and that end of life care is given due importance by the specialists caring for these patients often over long periods of time.

## STATE AND NATIONAL NETWORKS

Palliative care emphasizes provision of care for patients wherever they are. The Strategic Framework for Palliative Care in Victoria, Australia states as Principle 5 *“People with a life-threatening illness and their carers and families have treatment and care that is coordinated and integrated across all settings.”* Therefore, networks of care spanning hospitals and the community are needed.[2 3] These networks may be formed by one service working across all sites of care, by formal partnerships underpinned by contractual agreements or by informal working relationships which are based on collegiality and common goals of care. In Victoria, there are combinations of all types of relationships. A State government initiative has been to develop consortia, both metropolitan and regional, to assist in the delivery of coordinated care and help individual services to maintain quality of care through combined education, regional service development, and strategic allocation of resources (staff, equipment, specialist services, etc). Such networks take time to develop and require dedicated leadership time. Appointing clinicians or managers who already have fulltime commitments to be consortium leaders do not allow adequate time to achieve the aims of such networking. In addition, linking services that may previously have been competing in their region for sparse resources requires acknowledgment of and sensitivity to pre-existing relationships and some patience in establishing new relationships.

Another type of network is the clinical network. These groups of motivated clinicians, brought together by state or federal government, focus on the delivery of care and the quality of that care. Networks develop guidelines to assist services to implement care based on best available evidence. At times, networks set out to develop that evidence and trial the implementation strategies. Such a network has been created in Victoria to advise the Victorian Department of Health on the assessment of quality care in palliative care and to oversee the clinical elements and implementation of key initiatives for palliative care in Victoria. One initial Palliative Care Clinical Network project is to review the evidence for pain clinical indicators and recommend a set of indicators for palliative care services to collect and report. Another project is the review of palliative care clinical tools and outcome measures in order to make recommendation for introducing more consistent use of clinical tools across the state.

Finally, another emerging form of networking involves international collaborations. For example, Project Hamrahi is a collaboration between Australasian Palliative Link International (APLI) and Pallium India, developed to assist new services in India in their development of palliative care. The focus is particularly on cancer centers. Volunteer doctors and nurses in Australia are linked to a new service provider in India and will form lasting relationships through repeated visits, email and information sharing, and other creative ways to share knowledge and support. Other useful and important international collaborations exist in research and education but are outside the focus of this paper.
